# Combinations in multimodality treatments and clinical outcomes during cancer

**DOI:** 10.3892/ol.2020.12083

**Published:** 2020-09-09

**Authors:** Xiao-Ying Zhang, Pei-Ying Zhang

Oncol Lett 12: 4301-4304, 2016; DOI: 10.3892/ol.2016.5242

Following the publication of this article, an interested reader drew to our attention that the flow of the text, and the wording of numerous passages of text within the article, bore striking and unexpected similarities to the wording employed in a PhD thesis by Mi Yu at the National University of Singapore in 2013 entitled “Vitamin E TPGS Based Nanomedicine For Multimodality Treatment Of Cancer”.

After having conducted an internal investigation, the Editor of *Oncology Letters* has determined beyond all reasonable doubt that the review submitted to the Journal shared an unacceptable level of identity with the PhD thesis by Mi Yu. The authors were contacted about these apparent similarities between their review and the thesis, although they failed to respond to our query in this regard. The Editor of *Oncology Letters* deeply regrets that this article was not intercepted by our own checks for plagiarism prior to the publication of the review, and sincerely apologizes to the author of the PhD thesis and the readership for the inconvenience caused. We also thank the reader for bringing this matter to our attention.

